# Metabolic Reprogramming in Cancer: Role of HPV 16 Variants

**DOI:** 10.3390/pathogens10030347

**Published:** 2021-03-16

**Authors:** Adán Arizmendi-Izazaga, Napoleón Navarro-Tito, Hilda Jiménez-Wences, Miguel A. Mendoza-Catalán, Dinorah N. Martínez-Carrillo, Ana E. Zacapala-Gómez, Monserrat Olea-Flores, Roberto Dircio-Maldonado, Francisco I. Torres-Rojas, Diana G. Soto-Flores, Berenice Illades-Aguiar, Julio Ortiz-Ortiz

**Affiliations:** 1 Laboratorio de Biomedicina Molecular, Facultad de Ciencias Químico Biológicas, Universidad Autónoma de Guerrero, Av. Lázaro Cárdenas S/N, Ciudad Universitaria, Colonia La Haciendita, Chilpancingo C.P. 39090, Guerrero, Mexico; adanarizmendi@uagro.mx (A.A.-I.); mamendoza@uagro.mx (M.A.M.-C.); zak_ana@yahoo.com.mx (A.E.Z.-G.); trisrael5@yahoo.com.mx (F.I.T.-R.); diana.sotof@hotmail.com (D.G.S.-F.); b.illadesaguiar@gmail.com (B.I.-A.); 2 Laboratorio de Biología Celular del Cáncer, Facultad de Ciencias Químico Biológicas, Universidad Autónoma de Guerrero, Av. Lázaro Cárdenas S/N, Ciudad Universitaria, Colonia La Haciendita, Chilpancingo C.P. 39090, Guerrero, Mexico; nnavarro@uagro.mx (N.N.-T.); monseolea@uagro.mx (M.O.-F.); 3 Laboratorio de Investigación en Biomoléculas, Facultad de Ciencias Químico Biológicas, Universidad Autónoma de Guerrero, Av. Lázaro Cárdenas S/N, Ciudad Universitaria, Colonia La Haciendita, Chilpancingo C.P. 39090, Guerrero, Mexico; wences2009@hotmail.com (H.J.-W.); dinomtzcar@outlook.com (D.N.M.-C.); 4 Laboratorio de Investigación Clínica, Facultad de Ciencias, Químico Biológicas, Universidad Autónoma de Guerrero, Av. Lázaro Cárdenas S/N, Ciudad Universitaria, Colonia La Haciendita, Chilpancingo C.P. 39090, Guerrero, Mexico; dircio.mr@gmail.com; 5 Laboratorio de Diagnóstico e Investigación en Salud, Facultad de Ciencias Químico Biológicas, Universidad Autónoma de Guerrero, Av. Lázaro Cárdenas S/N, Ciudad Universitaria, Colonia La Haciendita, Chilpancingo C.P. 39090, Guerrero, Mexico

**Keywords:** HPV 16 variants, metabolic reprogramming, cancer

## Abstract

Metabolic reprogramming is considered one of the hallmarks in cancer and is characterized by increased glycolysis and lactate production, even in the presence of oxygen, which leads the cancer cells to a process called “aerobic glycolysis” or “Warburg effect”. The E6 and E7 oncoproteins of human papillomavirus 16 (HPV 16) favor the Warburg effect through their interaction with a molecule that regulates cellular metabolism, such as p53, retinoblastoma protein (pRb), c-Myc, and hypoxia inducible factor 1α (HIF-1α). Besides, the impact of the E6 and E7 variants of HPV 16 on metabolic reprogramming through proteins such as HIF-1α may be related to their oncogenicity by favoring cellular metabolism modifications to satisfy the energy demands necessary for viral persistence and cancer development. This review will discuss the role of HPV 16 E6 and E7 variants in metabolic reprogramming and their contribution to developing and preserving the malignant phenotype of cancers associated with HPV 16 infection.

## 1. Introduction

Cancer is a neoplasm that involves the alteration of at least six biological capacities: resisting cell death, sustaining proliferative signaling, evading growth suppressors, activating invasion and metastasis, enabling replicative immortality, inducing angiogenesis, and two emerging hallmarks—evading immune destruction and reprogramming energy metabolism, related to tumor initiation and progression [1]. In cancer, metabolic reprogramming promotes the energy increase necessary for growth, cell division, migration, and metastasis [2,3]. Otto Warburg was the first to propose that, unlike normal adult tissues, cancer cells produce energy mainly through aerobic glycolysis rather than oxidative phosphorylation, a state known as “aerobic glycolysis” or Warburg effect [2,4]. The Warburg effect favors obtaining adenosine 5’-triphosphate (ATP) necessary for cancer proliferation and metastasis by aerobic glycolysis [5,6].

Approximately 16% of cancer cases worldwide are caused by an infectious agent, mainly due to infection by oncogenic viruses [7] and about 5% are caused by high-risk human papillomavirus (HR-HPV) [8]. HR-HPV 16 is the leading cause of more than half of cervical cancer cases globally [9] and has been found in 50–55% of invasive cervical cancer cases [10]. It has also been linked to cancers such as head and neck, vulva, vagina, penis, and anal [11]. Currently, several reports show that human papillomavirus (HPV) 16 variants alter the expression of various genes related to apoptosis, adhesion, metastasis, angiogenesis, and metabolism [12]. These variants interact with multiple cellular proteins such as p53, retinoblastoma protein (pRb), p300/CBP, and pyruvate kinase 2 (PKM2), promoting gain or loss of their functions [13]. These have also been associated with a more significant oncogenic potential of these variants and the severity of precancerous lesions [14,15]. Additionally, it has been observed that E6 variants increase the expression of genes related to metabolic processes [16] and regulate signaling pathways related to glucose metabolism [17]. This review addresses the oncogenic role of HPV 16 variants in metabolic reprogramming modulation as a possible mechanism involved in carcinogenesis.

## 2. Energy Metabolism

Cellular energy metabolism is the bioprocess responsible for converting nutrients such as carbohydrates, lipids, and proteins into energy and biomass to maintain cell survival and proliferation [5]. Glucose is the main energy source for mammalian cells [5]. The entry of glucose to the cell is through solute transporters (SLC): Na^+^/Glucose cotransporters (SGLTs) and glucose transporters (GLUTs) [18]. Once glucose is internalized through the cell membrane by GLUTs or SGLTs, glycolysis begins, which represents one of the main ATP synthesis pathways. Physiologically, glycolysis occurs in the absence or presence of oxygen (O_2_) [19]. Enzymes involved in glycolysis are hexokinase (HK), glucose-6-phosphate isomerase (PGI), phosphofructokinase (PFK), aldolase (ALDO), triose phosphate isomerase (TPI), glyceraldehyde-3-phosphate dehydrogenase (GAPDH), phosphoglycerate kinase (PGK), phosphoglycerate mutase (PGM), enolase (ENO), and pyruvate kinase (PK) [19]. The final product of glycolysis under normal oxygen conditions is pyruvate, which is converted into acetyl-coenzyme A (acetyl-CoA) by the enzymatic complex pyruvate dehydrogenase (PDH), to be oxidized to CO_2_ and H_2_O through the cycle of tricarboxylic acids (TCA) and the electron transport chain (ETC) [20]. However, in hypoxia, pyruvate is converted to lactate by the enzyme lactate dehydrogenase A (LDHA) [21].

On the other hand, lipids also produce acetyl-CoA from the β-oxidation of fatty acids obtained from lipolysis or the diet [22]. Furthermore, many of the carbon skeletons of non-essential amino acids are intermediates of glycolysis and the TCA cycle used as energy sources [23]. The entry of acetyl-CoA to the TCA cycle is connected to cellular respiration, which integrates different energy sources derived from the diet, such as glucose, glutamine, and fatty acids [24]. The TCA cycle produces metabolic intermediates that are used as precursors for the synthesis of macromolecules, such as citrate in the synthesis of fatty acids and α-ketoglutarate, succinyl-CoA, and fumarate in the synthesis of amino acids; malate in gluconeogenesis; and electron acceptors that are used in processes such as the electron transport chain (ETC) [24]. Biochemical reactions in energy metabolism are regulated by various mechanisms involving the availability of substrates and allosteric regulation, which allows the cell to control energy production according to its energy state through the NADH/NAD+ ratio, ATP, and nutrients availability [24,25]. However, since 1930 it has been described that the normal conditions of energy metabolism are affected in cancer by metabolic reprogramming [26].

## 3. Metabolic Reprogramming in Cancer

Metabolic reprogramming is one of the critical hallmarks in cancer progression [27]. It is characterized by an increase in glycolysis to satisfy energy demand, anabolic, biosynthetic, and growth requirements, which promote the hyper-proliferation of cancer cells [28]. The absence of nutrients and O_2_ and secretion of secondary metabolites and carbon dioxide promote the formation of new blood vessels associated with the tumor, mainly activated by growth factors and tumor progression [29]. The best-known angiogenic regulators in cancer are vascular endothelial growth factor A (VEGF-A), von Willebrand factor (VWF), and thrombospondin-1 (TSP-1) [27,30]. In cancer models, it has been observed that angiogenesis is active from the early stages of tumor development to terminal stages. Besides, the regulation of angiogenesis and the supply of nutrients to satisfy energy demands and the excretion of metabolites are a limiting steps in developing tumors [29].

Since 1930 it was observed that, unlike normal cells, cancer cells show increased glycolysis even in the presence of O_2_; this process has been called “aerobic glycolysis” or “Warburg effect” [27]. Currently, it is known that the decrease in the incorporation of pyruvate in the mitochondria is the product of the high supply of glucose, glycolysis and of processes such as glutaminolysis, which is necessary for the synthesis of amino acids [3,28]. Furthermore, it has been observed that this metabolic reprogramming increases the expression of glucose transporters, especially GLUT1 and GLUT3, substantially increasing the importation of glucose into the cytoplasm of the cell [31,32,33]. Increased glycolysis in cancer cells is associated with the activation of oncogenes such as RAS and MYC or the silencing of tumor suppressor genes such as TP53 [3,32,34,35]. Similarly, an increase in the expression of glycolytic enzymes related to the Warburg effect such as HK2, PFK2, ALDO, GAPDH, PGK1, ENO1, PKM2, and LDHA has been observed in different cancers. In liver cancer cell lines such as Hep3B, HepG2, JHH5, JHH7, and Huh7, the expression of HK2 has been observed, while in other cancers such as lung, breast, colon, and also in HLF and HH6, liver cancer cell lines are expressed as both HK2 and HK1 [36]. The enzyme 6-phosphofructo-2-kinase/fructose-2,6-bisphosphatase (PFKFB or PFK-2) has been reported to be overexpressed in most tumor cells under hypoxic conditions [37], where it is responsible for maintaining cellular levels of fructose-2,6-bisphosphate (F-2,6-BP) by synthesizing or degrading fructose-2,6-bisphosphate. The kinase domain synthesizes F-2,6-BP from F-6-P and ATP. Simultaneously, the phosphatase site degrades F-2,6-BP to F-6-P and inorganic phosphate (Pi), such that the F-2,6-BP metabolite is regarded as the most potent allosteric activator of PFK-1 and an inhibitor of fructose-1,2-bisphosphatase [38]. ALDOA overexpression has also been found to be related to the Warburg effect in oral squamous cell carcinoma, osteosarcoma, hepatocellular cell carcinoma, and lung cancer. The overexpression of ALDOB plays an important role during the epithelial–mesenchymal transition. Likewise, the overexpression of ALDOC has been correlated with neuronal damage, such as schizophrenia, Alzheimer’s disease, and traumatic brain injury [39]. Furthermore, ALDOA has been proposed as a potential biomarker and molecular target for the early detection of hepatocellular carcinoma [40]. GAPDH also plays a key role in maintaining the Warburg effect in various cancers, such as lung, prostate, and pancreatic adenocarcinoma [41,42,43]. Increased GAPDH expression is also characteristic of cervical carcinoma tissue cells. However, it is not yet related to conventional clinicopathological parameters, such as clinical stage, histological type, or differentiation degree [44]. Until now, GAPDH was considered a target in the metabolism of glucose in liver cancer via the coactivator-associated arginine methyltransferase 1 (CARM1), which methylates GAPDH at arginine 234 (R234), inhibiting its catalytic activity and decreasing the proliferation of tumor cells in vitro and in vivo [45]. In aerobic glycolysis, another of the glycolytic enzymes that are altered in cancer cells to promote and maintain the Warburg effect is the PGK1 enzyme, which has been observed to be overexpressed in breast, colon, and gastric cancer [46,47,48]. Furthermore, different variants of PGK1 that differ in structure and function and are related to differentiation in catalytic efficiency or thermodynamic stability and changes in their tertiary structure have been identified in tumors. It is currently known that PGK1 is regulated by the transcription factor HIF-1, which promotes its overexpression and increases glycolysis [49,50]. Enolase, specifically isoform 1 (ENO1), has been reported as a critical regulator in tumor formation since the silencing of ENO1 has implications in the adaptation of autophagy and catabolic pathways, which affects the growth of tumors, inducing senescence [51]. PKM2 has also been reported to be overexpressed in cancer cells. However, it has been observed that it is not the only isoform of PKM overexpressed in cancer [52]. Similarly, the regulation of PKM2 in cancer is important for controlling cell metabolism and tumor growth [53]. PKM2 expression has been reported to increase due to alternative splicing, controlled by members of the heterogeneous nuclear ribonucleoprotein (hnRNP) family: hnRNPA1, hnRNPA2, and polypyrimidine tract binding protein (PTB; also known as hnRNPI). The transcription factor and oncoprotein c-Myc has also been observed to regulate increased expression of the three hnRNPs, thus contributing to PKM2 expression in some cancers [52]. PKM2 has been proposed to fluctuate between an active tetramer and a less active dimer. The PKM2 tetramer has a high affinity for phosphoenolpyruvate (PEP) and favors pyruvate and ATP production. In cancer cells, active PKM2 is present if glucose is used for energy production or the synthesis of anabolic precursors such as amino acids, nucleic acids, and phospholipids [52]. LDHA, an isoform of LDH, is predominantly expressed in cancer cells as a transcriptional regulation product and is activated by post-translational modifications such as phosphorylation and acetylation to increase lactate production during tumor progression and metastasis in cancer [54]. In metabolic reprogramming, tumor cells can metabolize lactate as an energy source that can be transported to neighboring cancer cells, adjacent stroma, and vascular endothelial cells. Lactate also plays a key role in promoting tumor inflammation and angiogenesis [55].

It is currently known that O_2_ concentrations are significantly reduced in many human cancers compared to normal tissue [56]. Median pO_2_ in breast cancer is ~10 mm Hg compared to ~65 mm Hg in normal breast tissue [56]; pO_2_ in tumor tissue is 0–20 mm Hg compared to normal tissue where it is 40 mm Hg [57]. The decrease in pO_2_ in cancer is associated with an increased risk of tumor invasion and metastasis [58]. The low availability of O_2_ in solid tumors is observed at a distance of approximately 100 µm from the blood vessels [59] due to hypoxic stress, favoring the activation of HIF1. Active HIF1 regulates the transcription of many genes such as TF (transferrin), HK2 (hexokinase 2), JMD1A (Jumonji domain-containing), CDH (cadherin), GLUT1, ALDO-C, LDH-A, PKM2, anhydrase carbonic IX (CAIX), ENO3,2, and PFKFB4 [60,61,62] that code for cancer-related proteins such as angiogenesis [63], genetic instability [64], invasion and metastasis [65,66], proliferation, glucose metabolism, and pH regulation [67,68,69]. HIF1 is composed of two subunits, the alpha and beta subunits (HIF-1α and HIF-1β) [70]; both subunits form the active dimer, necessary for the transcription of their target genes. The active HIF1 complex binds to DNA at specific sites called hypoxia response elements (HRE) in an RCGTG sequence. HIF-1β is constitutively expressed, while HIF-1α has a half-life of 5–8 min, which is increased under hypoxic conditions [71,72,73]. Under normal O_2_ conditions, HIF-1α is continuously expressed, but it is hydroxylated at two proline residues (P402 and P564) and one of asparagine (N803) by two dioxygenases, prolyl-4-hydroxylases (PHD) and asparaginyl hydroxylase or factor inhibitor HIF-1α (FIH-1), respectively [74]. These dioxygenases require O_2_, ferrous ion (Fe^2+^), and α-ketoglutarate (α-KG) for their activity and generate carbon dioxide and succinate as products [69]. Hydroxylated HIF-1α is ubiquitinated by the complex formed by von Hippel Lindau (VHL), Elongin C, Elongin B, Cullin 2 (CUL2), ring box protein 1 (RBX1), a ubiquitin E2 (E2) conjugating enzyme, and the spermidine/spermine protein N (1) -acetyltransferase 2 (SSAT2), to be subsequently degraded via the 26S proteasome [74]. Hydroxylation of the asparagine residue located in C-TAD inhibits the binding of HIF-1α to the transcriptional regulators CBP/p300; therefore, the transcriptional activity of HIF-1α is inhibited [75,76]. Under hypoxic conditions, the activity of PHDs and FIH1 on HIF-1α is affected, and metabolic reprogramming in a cancer cell is preferred [75]. The activity of PHDs and FIH1 also decreases in the absence of Fe^2+^ and α-KG. On the other hand, the loss of the function of succinate dehydrogenase (SDH) and fumarate hydratase (FH) favor the accumulation of succinate and fumarate, which bind to PHDs by inhibiting their activity; the binding of NAD(P)H: quinone oxidoreductase 1 (NQO1) to the ODDD of HIF-1α also inhibits the hydroxylation of HIF-1α. Likewise, the binding of the HSP90 chaperone in the domain Per-Arnt-SimA (PAS-A) of HIF-1α protects it from a degradation pathway independent of PHD/VHL, mediated by the activated receptor kinase C (RACK1) [75,77]. Stabilized HIF-1α is associated with polymerized microtubules and translocates to the nucleus when the motor protein dynein recognizes its nuclear localization signal (NLS). HIF-1α forms the active HIF1 complex in the nucleus to regulate the transcription of its target genes [61,62,77] by binding to DNA in HREs [72]. Active HIF1 promotes the expression of glucose transporters such as GLUT1 that increases the amount of glucose in the cytoplasm [32,33], oncogenes such as c-Myc, and glycolytic enzymes related to the Warburg effect such as HK2, PFKFB or PFK-2, ALDOA- B, GAPDH, PGK1, ENO1, PKM2, and LDHA-B. These HIF-1a target enzymes have also been observed in different types of cancer associated with high-risk human papillomavirus (HR-HPV), including HPV 16 [36,52,53,78,79,80,81].

## 4. Human Papillomavirus 16

The HR-HPV that causes more than half of cervical cancer cases in the world is HPV 16 [9]; it has been found in 50–55% of invasive cervical cancer cases [10], and it is suggested it has a biological advantage for viral transmission and persistence, as well as cell transformation [82]. Infection by HPV 16 requires micro-lesions in the host for the virions to access the cells of the basal layer. In these cells, considered as the infection reservoir, the viral genome remains as an episome with a low number of viral copies. The cells of the intermediate layers of the epithelium where HPV E6 and E7 are expressed promote cell transformation. The E6 oncoprotein interacts with multiple cellular proteins, affecting their function or promoting their degradation. For example, the interaction with ubiquitin ligase E6AP and subsequent degradation of p53 inhibits apoptosis, keratinocyte differentiation, and the response to interferons. Likewise, it has been observed that E6 activates factor c-Myc, telomerase hTERT, and the signaling pathways AKT, Wnt, Notch, and mTORC1 [83,84,85].

On the other hand, E7 binds to the proteins pRB, p107, and p130, promoting their degradation, which favors the progression of the cell cycle, stability and amplification of the genome, immortalization, and cellular transformation. Likewise, it suppresses the function of STAT1, activates the AKT signaling pathway, and binds to other proteins such as E2F1, Cullin2, and HDAC [83,84,85]. The viral cycle is completed when the viral genome is encapsulated and immature virions are released into the upper layer of the epithelium [86]. Furthermore, it is related to other cancers, such as head and neck, vulvar, vaginal, penile, and anal cancer [11]. It has been shown that the E6 and E7 oncoproteins are overexpressed in cells infected with HPV favoring tumorigenesis. Variants of the HPV 16 E6 and E7 oncoprotein have also been reported to regulate the expression of multiple genes involved in various cellular processes such as proliferation, differentiation, apoptosis, adhesion, angiogenesis, transcription, and protein translation [12,87,88].

### Variants of HPV 16

The classification of HPV 16 variants has been made according to the distribution by their geographic origin [89]. In the analysis of their genome, the HPV 16 variants have been grouped into four main phylogenetic lineages: A (European-Asian, EAS), B (African 1, AF1), C (African 2, AF2), and D (North American/Asian American, NA/AA) [90]. The four lineages, in turn, have been subdivided into eleven sublineages: (1) A1, A2, A3 (European, E), A4 (Asian, As); (2) B1 (Afr1a), B2 (Afr1b); (3) C1 (Afr2a), C2 (Afr2b); and (4) D1 (North American, NA), D2 (Asian American 1, AA1), and D3 (Asian American 2, AA2) [90,91]. The sublineages have also been stratified into classes and subclasses [92]. The study of the variants of the specific genes E6 and E7 of HPV 16 has shown that the most frequent E6 variant is E-G350. However, AA-a showed a higher risk of developing cervical cancer, followed by E- A176/G350, AA-c, E-G350, and E-C188/G350 compared to the E-prototype [93]. On the other hand, the E7 variants have been studied to a lesser extent than the E6 variants. Some studies have reported, based on the conserved regions (CR) 1, CR2, and CR3 of E7, 4 variants in the CR1 domain [94,95], 5 in CR2 [94,96,97,98,99], and 14 in CR3 [94,95,100,101,102,103]. The most frequent variant, up to 70% in the Asian population, is the E7-G647 variant located in the CR2 domain [88,94].

## 5. Mechanisms Involved in HPV 16 Variants-Mediated Metabolic Reprogramming

Variants of HPV 16 have been associated with the oncogenic potential of the virus and the severity of cervical lesions [14,15]. It has been reported that the E6 and E7 oncoprotein of HR-HPV can promote metabolic reprogramming through increased activity of glycolytic enzymes and inhibition of the Krebs cycle and the respiratory chain to satisfy the energy requirements of cancer cells and achieve efficient viral replication [81,104]. Martínez-Ramírez and collaborators describe the enzymes and metabolic pathways altered by HR-HPV and report that HPV 16 increases glycolysis in oral squamous cell cancer. In lung cancer, GLUT1 transporter levels increase; in cervical cancer, glycolysis is due to increased glycolytic enzymes HKII, PFK, ENOA, PKM2, and LDHA. In contrast, oxidative phosphorylation (OXPHOS) is affected by HPV 16 in cervical cancer by modulating the mitochondrial structure and the release and increase of reactive oxygen species (ROS). This finding is explained by the inactivation of mitochondrial Complex III and ATP synthase and a decrease in glutathione (GSH) and superoxide dismutase 1 and 2 (SOD1 and 2) levels [81]. Additionally, it has been observed that E6 variants increase the expression of various genes, of which more than 60% correspond to genes associated with metabolic processes. [16]. The possibility exists that variants of the E6 and E7 oncoprotein of HPV 16 may favor metabolic reprogramming. Next, we will describe the role of HPV 16 variants in regulating the molecular mechanisms of cell metabolism in cancer.

### 5.1. p53 Degradation

**Figure 1:**
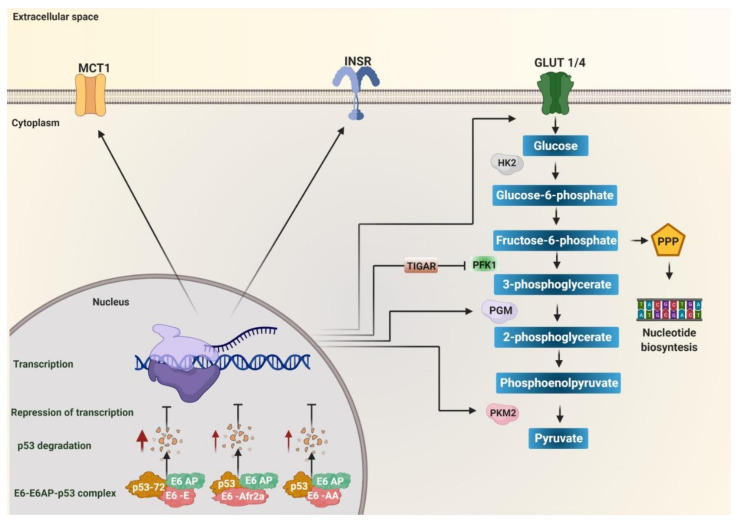
The degradation of p53 induced by E6 variants of human papillomavirus (HPV) 16 favors metabolic reprogramming. Variants of the European (E), African 2a (Afr2a), and Asian American (AA) sublineages decrease p53 levels through interaction with E6AP, increasing p53 degradation (red arrows), and avoiding transcriptional repression. p53 inhibits glycolysis by repressing transcription of the monocarboxylate transporter 1 (MCT1), the glucose transporters GLUT1 and GLUT4, the enzymes hexokinase 2 (HK2), phosphoglycerate mutase (PGM), pyruvate kinase 2 (PKM2), and TP53 induced glycolysis regulatory phosphatase (TIGAR). TIGAR decreases the activity of the enzyme phosphofructokinase 1 (PFK1) to target fructose-6-phosphate to the pentose phosphate (PPP) pathway. Insulin receptor (INSR) favors the translocation of GLUT4 to the membrane. Therefore, the absence of p53 due to its degradation by the effect of the variants of the E, Afr2a, and AA sublineages promotes glycolysis by activating the expression of the transporters GLUT1, GLUT4, MCT1, and the enzymes of the glycolytic pathway HK2, PGM, PKM2. Additionally, it targets fructose-6-phosphate to the PPP pathway via TIGAR for nucleotide synthesis.

Metabolic reprogramming in cancer is carried out by different molecular mechanisms related to p53, MYC, or HIF-1α [105]. In recent years, variants of the E6 oncoprotein of HPV 16 have shown differences in their ability to bind p53 and induce its degradation [106], through the stability of the E6-E6AP-p53 complex [107]. HPV 16 variants AA, E, and Afr2a have been shown to reduce p53 levels in human keratinocytes (Figure 1), exhibiting a more significant effect than the E-prototype and E-G131 (R10G) variants [108,109,110,111]. The effect of the E-prototype variant on p53 degradation has been related to the polymorphism in codon 72 of p53, which results in an arginine genotype (CGC), conferring p53 greater susceptibility to being degraded by E6 (Figure 1) [112]. Higher expression of European E6 sublineage variants has also been reported to be related to greater p53 degradation compared to As sublineage variants [109]. Additionally, in a group of patients with cervical cancer, the presence of the polymorphism in codon 72 (homozygous arg/arg genotype of p53) with the E-prototype variant was related to the clinical status and the susceptibility for p53 to be degraded [113].

P53 is currently known to regulate glycolysis and oxidative phosphorylation (OXPHOS) [114]. It has been observed that it inhibits the entry of glucose into the cell by directly repressing the transcription of the glucose transporters GLUT1 and GLUT4 [115], in addition to inhibiting the translocation of GLUT1 by directly inducing the transcription of the Ras-related glycolysis inhibitor and calcium channel regulator (RRAD) [116]. It can also inhibit glycolysis by repressing the insulin receptor (INSR) expression, necessary in the translocation of GLUT4 to intracellular vesicles that are subsequently directed to the plasma membrane and, in turn, triggers a rapid increase in glucose absorption [117]. It negatively regulates HK2 and activates the expression of TP53 induced glycolysis regulatory phosphatase (TIGAR), reducing glycolytic activity and attenuating the activity of PFK1, leaving glucose-6-phosphate as a necessary substrate in the nucleotide synthesis in the pentose phosphate pathway [118,119]. Furthermore, it can inhibit glycolysis by reducing the expression of the glycolytic enzyme phosphoglycerate mutase (PGM), responsible for converting 3-phosphoglycerate (3PG) to 2-phosphoglycerate (2PG) during glycolysis [120] or by decreasing PKM2 (via the ubiquitin ligase Parkin, responsible for ubiquitinating PKM2) [114]. P53 has also been shown to inhibit lactate transport by repressing monocarboxylate transporter 1 (MCT1), causing lactate to accumulate and limit the glycolytic rate in cancer cells [121]. P53 positively regulates the entry of pyruvate into the mitochondria and the progression of TCA by increasing the amount of pyruvate carboxylase [122]. It has also been reported that p53 represses the function of transcription factors that induce the transcription of glycolysis regulatory enzymes, such as factor HIF1 [114]. It has been observed that p53 binds to the HIF-1α/p300 complex to decrease its transcriptional activity; likewise, it also negatively regulates c-Myc [123,124]. In summary, the E6 variants of HPV 16 of the AA, E, and Afr2a sublineages can alter glucose metabolism by inducing p53 degradation, leading to increased expression of enzymes, transporters, and receptors involved in glycolysis such as GLUT1, GLUT4, INSR, HK2, TIGAR, PFK1, PKM2, MCT1, pyruvate carboxylase (PC) (Figure 1), and the positive regulation of the transcription factors HIF-1α and c-Myc related to the transcription of genes involved in the glycolysis. Thus, the E6 variants of HPV 16 that induce a more significant degradation of p53 favor the increase of glycolysis in HPV-associated cancers.

### 5.2. pRb Degradation

**Figure 2:**
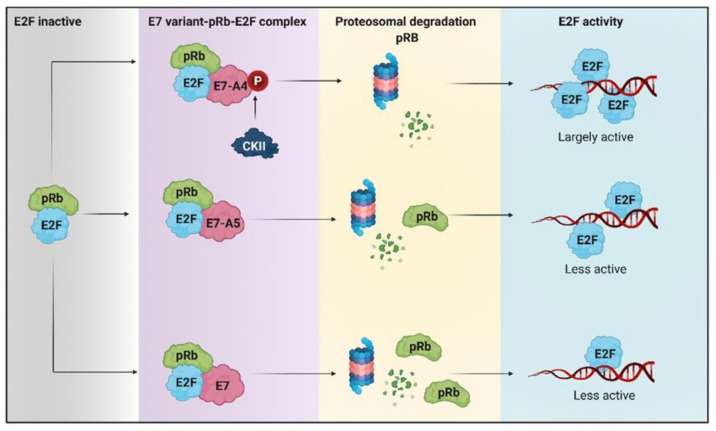
The degradation of retinoblastoma protein (pRb) induced by HPV 16 E7 variants favors metabolic reprogramming. The hyperphosphorylated pRb repressor allows the release of E2F. The E7-A4 variant has a new phosphate acceptor site for casein kinase II (CKII) that promotes more significant degradation of pRb, compared to A5 and other E7 variants that degrade pRb to a lesser extent. Therefore, there is greater activation of E2F by the A4 variant than different E7 variants in which E2F is less active.

It is currently known that in cancer cell lines, the HPV 16 E7 oncoprotein is located preferentially in the nucleus and, to a lesser extent, in the cytoplasm, which is related to the decrease in retinoblastoma protein (pRb) levels [125]. Furthermore, dephosphorylated pRb has been reported to bind to the transcription factor E2F, inhibiting its transcriptional activity [126]. Factor E2F is activated when pRb is phosphorylated as it allows its release from the pRb/E2F complex, thus binding to promoters of genes essential for cell cycle progression cdc2 and cyclin E [126,127]. Recently it has been reported that the variant A4 that has the N29S mutation in the E7 oncoprotein promotes more significant degradation of pRb compared to the variant designated A5 that possesses the L28F mutation (Figure 2) [128]. It has been reported that the mutation in variant A4 generates a new phosphate acceptor site for casein kinase II (CKII) that confers a greater phosphate-dependent interaction for pRb and that leads to its degradation in a greater proportion, compared to other E7 variants (Figure 2) [88,129]. The lack of pRb in tumors reduces oxidative metabolism, even when ATP levels do not decrease, increases the use of glutamine as a carbon source for obtaining energy, and increases the expression of genes involved in the synthesis of nucleotides such as dihydrofolate reductase (DHFR), thymidylate synthase (TS), ribonucleotide reductase (RNR), and thymidine kinase (TK), which are induced by E2F [130]. Considering that the A4 and A5 variants of HPV 16 E7 cause pRb degradation, it is suggested that these variants may be affecting metabolism as an alternative route that will allow them to obtain the necessary energy to maintain the replicative state of tumor cells.

### 5.3. Activation of c-Myc and Expression of Its Target Genes

**Figure 3:**
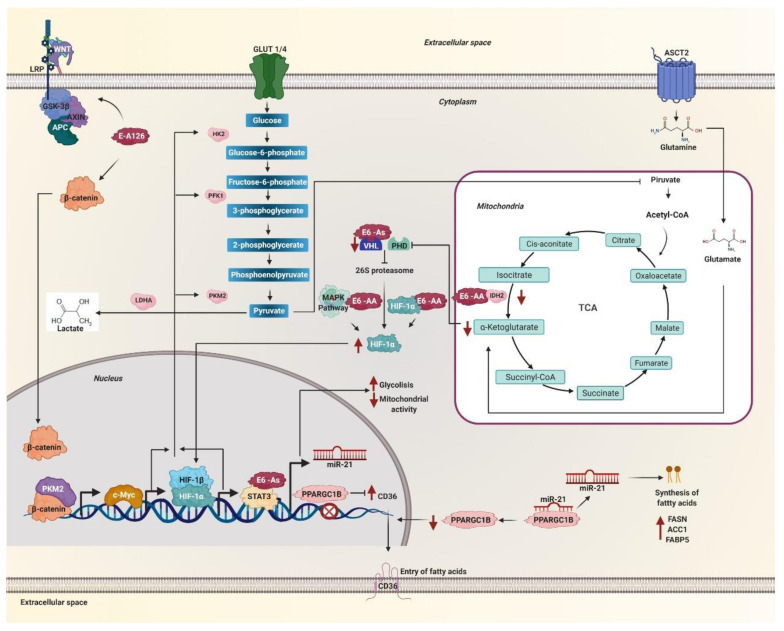
Metabolic reprogramming is mediated by HPV 16 E6 variants through c-Myc, hypoxia-inducible factor (HIF)-1α, WNT, and miR-21. The E6 variant E-A126 with the R8Q mutation promotes the glycolytic pathway by activating the Wnt/β-catenin pathway. Variants of the AA sublineage activate the mitogen-activated protein kinases (MAPK) pathway that increases HIF-1α levels. The protein β-catenin translocates to the nucleus accompanied by PKM2 and induces the expression of c-Myc. These variants also decrease the levels of the isocitrate dehydrogenase 1 (IDH2) enzyme necessary to obtain α-ketoglutarate, one of the substrates of the prolyl-4-hydroxylases (PHDs) enzymes. On the other hand, the As sublineage variants decrease the von Hippel Lindau (VHL) ubiquitin ligase levels, both enzymes are necessary for HIF-1α to be degraded via the 26S proteasome. Therefore, the variants of the AA and As sublineages increase the levels of HIF-1α, avoiding its degradation via the proteasome. Consequently, HIF-1α stabilizes and translocates to the nucleus. Both HIF-1α and c-Myc increase the levels of glycolysis enzymes such as hexokinase 2 (HK2), phosphofructokinase 1 (PFK1), pyruvate kinase M2 (PKM2), lactate dehydrogenase A (LDH-A), as well, lactate levels increase. The STAT3 activator increases glycolysis, decreases mitochondrial activity and allows the transcription of miR-21, which is also positively regulated by variants of the As sublineage. MiR-21 inhibits the PPARGC1B messenger. The levels of the CD36 transcriptional repressor PPARGC1B decrease and allow the expression and translocation of CD36. In this way, miR-21 regulates the entry and synthesis of fatty acids in the cell by the effect of the variants of the AA and As sublineages.

The transcription factor c-Myc is considered one of the most deregulated oncogenes in cancer [131]. It is known to be linked to alterations in cancer metabolism through the regulation of genes involved in the biogenesis of ribosomes and mitochondria, as well as genes involved in the metabolism of glucose, nucleotides, and glutamine [132]. The metabolic reprogramming induced by c-Myc is related to the increase of GLUT1, HK2, LDHA, PKM2, and ENO1 proteins of glycolytic metabolism and the increase of the glutamine transporters ASCT2 and glutaminase (GLS) enzyme (Figure 3) [133]. Under hypoxic conditions, c-Myc collaborates with active HIF1 by regulating pyruvate dehydrogenase kinase 1 (PDK1) levels, suppressing mitochondrial respiration, and promoting glucose conversion to lactate [132]. Variants exhibiting the R17I and Q21D mutations in the HPV 16 E6 oncoprotein have been shown to stabilize the E6-p53-E6AP complex [107]. Since the E6-p53-E6AP complex binds to the c-Myc protein [134], it is suggested that the variants of the E6 oncoprotein could be increasing the activity of c-Myc and the expression of its target genes involved in metabolism. On the other hand, the E-A126 variant that possesses the R8Q mutation in the E6 oncoprotein activates the Wnt/β-catenin signaling pathway [135] promoting that β-catenin and PKM2 are translocated to the nucleus, inducing the expression of c-Myc (Figure 3) [136]. Therefore, the variants present the R17I, Q21D, and R8Q mutations in the E6 oncoprotein of HPV 16 could be favoring metabolic reprogramming, increased nucleotide synthesis, and glutaminolysis through c-Myc, HIF-1α, and pyruvate dehydrogenase kinase 1PDK1.

### 5.4. Stability of HIF-1α and Expression of Active HIF-1 Target Genes

HPV 16 E6 has been reported to promote the hypoxia-induced Warburg effect by binding HIF-1α and VHL by blocking the interaction between HIF-1α and VHL [137]. Lack of interaction between HIF-1α and VHL attenuates VHL-mediated ubiquitination of HIF-1α, preventing degradation of HIF-1α, suggesting that HPV 16 E6 plays an essential role in the regulation of the Warburg effect [138]. A decrease in the enzyme isocitrate dehydrogenase 1 (IDH1) and IDH2 has been observed in response to the AA/E7-prototype variant compared to E-prototype/E7-prototype [137], and a decrease in the expression levels of the VHL ubiquitin ligase by the Asian variant that has the D25E mutation in the E6 oncoprotein (Figure 3) [16]. IDH is necessary for the production of α-KG, used for the hydroxylation of HIF-1α under normoxia conditions, while VHL is a fundamental part of the degradation of HIF-1α via the proteasome. Therefore, a decrease in IDH and VHL increases the stability of HIF-1α [139]. Variants of the AA sublineage of the HPV 16 E6 oncoprotein have been shown to increase HIF-1α levels in the keratinocyte nucleus under hypoxic conditions (Figure 3) [17]. According to the levels of HIF-1α induced by AA sublineage variants, an increase in transcriptional activity has been observed under hypoxic conditions [17]. GLUT1 levels in cancer significantly increase in cells with AA sublineage variants (Figure 3) [17,140,141]. Additionally, HIF-1α increases the levels of PKM2 and the transcriptional activator STAT3, which increases aerobic glycolysis and decreases mitochondrial activity (Figure 3) [142]. High levels and nuclear translocation of HIF-1α are related to the ability of AA sublineage variants to enhance mitogen-activated protein kinases (MAPK)/extracellular signal-regulated kinase (ERK)1/2 phosphorylation (Figure 3) [17]. These data together indicate that variants of the AA and As sublineages of the HPV 16 E6 oncoprotein positively regulate the Warburg effect by increasing levels of metabolic proteins and transcriptional factors STAT3 and HIF-1α.

### 5.5. Activation of Signaling Pathways That Regulate Glycolysis

Aerobic glycolysis in cancer has been reported to occur downstream of cellular signaling pathways [143]. Among the signaling pathways that regulate aerobic glycolysis in cancer are members of the family of mitogen-activated protein kinases (MAPK), which in turn is divided into three subfamilies of MAPK: extracellular signal-regulated kinase (ERK), the N-terminal c-Jun kinase (JNK), and the p38 kinase. Members of the three MAPK subfamilies have been shown to regulate the redirection in energy production from glycolysis in malignant and highly proliferative cells by affecting the activity of key metabolic regulators, necessary for energy production in the development of cancer [143]. The MAPK signaling pathway is activated by the E-G350 (L83V) variant of the E6 oncoprotein of HPV 16. It is related to larger and more aggressive tumors compared to the E-prototype variant [144]. Variants of the AA sublineage have been reported to exhibit more significant expression of the MAP2K1 gene [145] that codes for MAPK1, also known as MEK1; a component of the RAS mitogen-activated protein kinase (MAPK) pathway, MAPK1 is a threonine/tyrosine kinase that activates ERK1 and promotes autophagy [146]. Autophagy in tumor cells is necessary to prevent energy crisis and maintain nucleotide reserves during starvation [147] by recycling macromolecules, thus providing bioenergetic and biosynthetic substrates TCA cycle, which maintains energy homeostasis and nucleotide levels [148]. Autophagy is increased to promote survival, growth, and malignant neoplasia autonomously in tumor cells [148]. Variants of the AA sublineage have a greater capacity to activate the signaling pathway: MAPK-ERK, MAPK-p38, and PI3K/AKT (Figure 3) [149]. It has been observed that the E-G350 variant and the variants of the AA sublineage of the E6 oncoprotein of HPV 16 affect the regulation of metabolic reprogramming in cancer, the E-G350 variant through MAPK, and the variants of the sublineage AA by obtaining nutrients through MEK1/ERK1-activated autophagy and by activating MAPK-ERK, MAPK-p38, and PI3K/AKT signaling. The MAPK-p38 signaling pathway positively regulates GLUT1 and GLUT4 expression and glucose uptake [150,151]. The low activation of the AKT pathway is related to PKM2, ENO1, ENO2, and HK2 expression through the HIF-1/AKT signaling pathway [152]. On the other hand, it has been observed that the ERK and PI3K signaling pathways are necessary for the expression of LDH and pyruvate dehydrogenase kinase (PDK). However, the activation of PDK is also dependent on Src/JNK [153]. These data suggest that variants of the AA sublineage and the E-G350 variant of the E6 oncoprotein of HPV 16, both with the T350G mutation, play a crucial role in the reprogramming of glycolytic metabolism at the level of three critical components of pyruvate metabolism, LDH, pyruvate dehydrogenase (PDH), and PDK, in which the entry of pyruvate to the TCA cycle is inhibited by being metabolized to lactate.

The Wnt signaling pathway has been related to the formation of malignant tumors such as breast, colon [154], and cervical cancer, where the Wnt pathway has been suggested as one of the main dysregulation pathways [155]. The HPV 16 variants that present the E-A126 (R8Q), E-G131 (R10G), E-G350 (L83V), and E-T245 (R48W) mutations in the E6 oncoprotein. These can hyperactivate the Wnt signaling pathway, being activated more by the variant presenting the R8Q mutation (Figure 3), followed by the variants showing the L83V, R10G, and R48W mutations [135]. The E-A126 (R8Q) variant was found to exhibit a greater effect on the activation of β-catenin transcription (Figure 3) [135]. The Wnt/β-catenin pathway regulates the activation of the tyrosine kinase receptors that leads to the activation of the PI3K/AKT pathway, which leads to an increase in glucose metabolism, which in turn leads to the activation of the Warburg effect through HIF-1α, which in turn increases the expression of GLUT, HK, PKM2, LDH-A, and PDK, resulting in increased cytosolic pyruvate, of which most is converted to lactate and released to the extracellular environment, while a small part is converted to acetyl-CoA, which enters the TCA cycle and is converted to citrate to promote lipid and protein synthesis [136]. Furthermore, the Wnt/β-catenin pathway has been shown to increase the Warburg effect by suppressing mitochondrial respiration by reducing cytochrome oxidase transcription, an essential enzyme for OXPHOS [154]. On the other hand, AA sublineage variants increase mTOR activation, which, like the Wnt/β-catenin pathway, leads to energy metabolism activation through HIF-1α [13].

### 5.6. Overexpression of miR-21

MicroRNAs (miRNAs) are small RNAs of 20 to 24 nucleotides that control gene expression by binding to the 3’UTR, 5’UTR, or non-coding regions of messenger RNA (mRNA), which leads to degradation of mRNA or inhibition of protein synthesis [156,157]. Studies in cervical cancer cells transfected with variants of the Asian sublineage of the E6 oncoprotein of HPV 16 observed a 2- to 3-fold higher expression of miR-21 (Figure 3) compared to the E-prototype variant [158]. The increased expression levels of miR-21 have been associated with STAT3 expression, the transcriptional activator of miR-21 (Figure 3) [158]. miR-21 has also been reported to promote carcinogenesis by regulating fatty acid metabolism by promoting increased cellular lipid content, including cellular phospholipid content, neutral lipid content, and cellular triglyceride content [159], by increasing the expression of the lipid and fatty acid receptor CD36, since it targets PPARGC1B, a target of miR-21, a transcriptional repressor of CD36 [159,160]. Furthermore, miR-21 is involved in the increase of crucial lipid metabolic enzymes such as fatty acid synthase (FASN), acetyl-CoA carboxylase 1 (ACC1), and fatty acid-binding protein 5 (FABP5) [159]. However, the mechanism by which it favors the increase of these enzymes is still unknown. These data suggest that carcinogenesis promoted by the variants of the As sublineage is increased since they promote the expression of miR-21 favoring the entry and synthesis of fatty acids, necessary for the production of energy and synthesis of cell membranes, as well as the expression of key enzymes of lipid metabolism (Figure 3).

### 5.7. Regulation of Metabolic Enzymes

**Figure 4:**
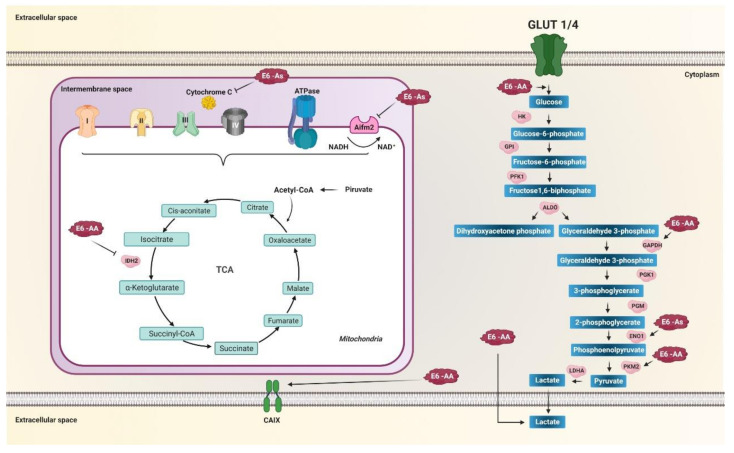
E6 variants of HPV 16 regulate the activity of enzymes related to metabolic reprogramming. Variants of the AA sublineage increase glucose entry into the cell, increase glyceraldehyde-3-phosphate dehydrogenase (GAPDH), PKM2, and increase lactate release. Variants of the As sublineage increase enolase 1 (ENO1) levels. Mitochondrial metabolism is decreased by variants of the AA and As sublineages. Variants of the AA sublineage decrease IDH2 in the tricarboxylic acid cycle (TCA), whereas variants of the As sublineage decrease AIFM2 and cytochrome c in the electron transport chain (ETC). Variants of the AA sublineage increase the carbonic IX (CAIX) enzyme levels, essential for balancing the pH of the tumor microenvironment induced by aerobic glycolysis. Arrows in black indicate activation and positive regulation as appropriate. The thick black arrows indicate the increase in the product of the enzymatic reaction. Arrows T indicate inhibition.

During the cell cycle, cells experience a series of events that require a significant supply of energy. It has been observed that during the G1 phase and the synthesis phase, or S phase, energy requirements are increased due to the synthesis of proteins, nucleic acids, and lipids. Therefore, glycolysis and pyruvate production is increased [161]. It has been reported that cultures of primary human foreskin keratinocytes (PHFK) transfected with the E7-prototype variant and those of the AA sublineage of the E6 oncoprotein of HPV 16 exhibit a more significant proportion of cells in the G1 phase, higher levels of enzymes of the metabolism of glucose, and low levels of TCA enzymes [137]. A decrease in the enzyme isocitrate dehydrogenase 1 and 2 (IDH1, IDH2) has also been observed in PHFK transfected with the bicistron containing the variant of the AA and E7-prototype sublineage (AA/E7-prototype) compared to the bicistron with the variants E-prototype/E7-prototype [137]. Likewise, higher GAPDH and PKM2 were observed in PHFK with AA/E7-prototype variants compared to E-prototype/E7-prototype (Figure 4), and low levels of phosphoglucomutase enzymes-2 and aldo-ketoreductase with the bicistron of the AA/E7-prototype variants compared to E-prototype/E7-prototype [137]. These data suggest that the oncogenicity of the variants of the AA sublineage of the E6 oncoprotein of HPV 16 is related to the Warburg effect by increasing glucose metabolism enzymes (Figure 4) [6] and decreasing enzymes of the tricarboxylic acid cycle as the IDH necessary for the production of α-ketoglutarate used in the hydroxylation of HIF-1α for its subsequent degradation via proteasome under normoxic conditions. In this sense, the decrease in IDH favors an increase in the stability of HIF-1α, allowing the expression of its target genes involved in glycolytic metabolism [139], a hallmark of cancer [6,27]. It was also reported that in PHFK with variants of the AA sublineage of the E6 oncoprotein of HPV 16, glucose entry into the cell increases (Figure 4). It was observed that PHFKs with variants of the AA sublineage are capable of internalizing up to 1.65 times more glucose compared to PHFK with the E-prototype variant, which is accompanied by an increase in glucose consumption and a greater secretion of lactate compared to PHFK containing the E-prototype (Figure 4) [17]. Lactate excretion into the extracellular space is considered part of the tumor microenvironment. It is related to extracellular proteolysis, which results in an acid-resistant phenotype necessary for cancer cell migration and metastasis [162].

On the other hand, AA sublineage variants increase the expression of CAIX compared to the E-prototype; this effect is greater in the presence of E7-prototype (Figure 4) [163]. The CAIX gene is highly expressed in response to hypoxia and is considered a general marker of tumor hypoxia, considered part of the tumor microenvironment in numerous solid tumors [164,165]. This suggests that the expression of CAIX is directly related to the oncogenic potential of the AA sublineage variants of the E6 oncoprotein, where there is a more significant increase compared to the E-prototype [163]. One of the ways that could also be participating in the positive regulation of glycolysis through the AA sublineage variant is by binding to the gamma-2 subunit of the protein kinase activated by 5’-AMP (PRKAG2) [13], a regulator key to vigorous metabolism that activates energy production pathways and inhibits energy-consuming processes [13].

On the other hand, the Asian variant of E6, containing the amino acid change D25E in the E6 oncoprotein, negatively regulates the expression of the AIFM2 protein [16], known as apoptosis-inducing factor mitochondria associated 2, which contains a domain oxidoreductase NADH [166]. It has also been observed that the variant of the AA sublineage binds to cytochrome C [13] and could be interfering at levels such as the Asian variant D25E [13,16,167], which also increases the expression levels of ENO1 (Figure 4) [16]. The low levels of AIFM2 and cytochrome C, as well as the increase in the expression of ENO1 induced by the variant AA and As, may affect the reduction of oxidative phosphorylation, favoring the production of energy through aerobic glycolysis, one of the main characteristics of metabolic reprogramming in cancer. Furthermore, the As variant has been shown to decrease the levels of the enzyme GSTP1 (glutathione S transferase Pi 1) [168], a critical cytosolic enzyme of phase II detoxification metabolism [169]. Low levels of GSTP1 are associated with DNA damage from reactive oxygen species and drug resistance in cancer [169], suggesting that the As variant of HPV 16 may negatively regulate phase II detoxification metabolism. Consequently, ROS levels and DNA damage that favors integrating the viral genome into the cellular genome can be increased.

The integration of the HPV 16 genome into the host genome is associated with carcinogenesis, not only because of the overexpression of E6 and E7 but also because it can induce the several host overexpression mRNAs [170]. As observed in epithelial samples with the integrated viral genome of the variants of the AA sublineage of the E6 oncoprotein of HPV 16, there is an overexpression of the SLC26A2 gene that encodes a solute transporter [171]. The SLC26A2 gene belongs to the family of SLC26 genes that encode solute transporters responsible for transporting sulfate (SO42^−^), Cl^−^, and oxalate [172]. Oxalate can be produced by epithelial cells and be present as free oxalic acid or in salts such as sodium, calcium, or potassium oxalates [173,174,175,176]. High oxalate levels may be related to the transformation of normal to tumor cells, increasing proliferation [177] and mitochondrial damage, as well as altering basal proton output and mitochondrial oxygen consumption linked to ATP production [178] through damage to mitochondrial proteins [179]. These data suggest that variants of the AA sublineage of the HPV 16 E6 oncoprotein might have a different effect on dysregulation of mitochondrial metabolism and increased aerobic glycolysis through integration and overexpression of SLC26A2. Table 1 summarizes the effect that the E6 and E7 variants of HPV 16 has on metabolic reprogramming in cancer.

## 6. Conclusions

Metabolic reprogramming is one of the hallmarks of cancer. There is a considerable increase in glycolysis and lactate production even in the presence of oxygen, a phenomenon known as the “Warburg effect”. Likewise, there is an increase in the synthesis of fatty acids and glutaminolysis. The E6 and E7 oncoproteins of HPV 16 can promote metabolic reprogramming through increased activity of glycolytic enzymes and decreased oxidative metabolism to meet the energy requirements of cancer cells and achieve efficient viral replication. The E6 and E7 variants of HPV 16 present a different oncogenic potential for cancer development through various mechanisms that promote metabolic reprogramming. The variants of the E, As, AA, and Afr2a sublineages, which present the R10G, R48W, D25E, Q14H, H78Y, L83V, R8Q, R17I, and Q21D mutations of the E6 oncoprotein of HPV 16 favor metabolic reprogramming, increasing the entry of glucose and glycolysis due to the increase in the levels of GLUT1 and the enzymes of the glycolytic pathway such as HK2, GAPDH, ENO1, PKM2, LDH-A, decreasing oxidative metabolism due to the decrease in the levels of proteins such as PDK, IDH1, IDH2, Aifm2, and cytochrome C, likewise stimulating the glutaminolysis pathway by increasing the ASCT2 transporter and the GLS enzyme, as well as the entry and synthesis of fatty acids, necessary for energy production and synthesis of cell membranes, processes mediated by the degradation of p53, increased levels of c-Myc and the stability of HIF-1α, activation of the Wnt pathway, and overexpression of miR-21.

On the other hand, the E7 A4 variant promotes metabolic reprogramming by increasing glutaminolysis and nucleotide synthesis through pRb degradation and E2F activation. The mechanisms described show that HPV 16 variants regulate metabolism in cancer through metabolic reprogramming; however, further research is necessary to understand how variations in the nucleotide and amino acid sequence of E6 and E7 of HPV 16 may be favoring metabolic reprogramming. It is required to carry out more detailed analyses on the interaction of the variants of the E6 and E7 oncoproteins of HPV 16 with proteins involved in altered metabolic pathways during metabolic reprogramming to understand the development of this pathology and to be able to propose new markers and strategies in the HPV 16 cancer therapy. The information presented in this review shows that the E6 and E7 variants of HPV 16 play a crucial role in metabolic reprogramming, and the detection and identification of the viral type are of great importance in patients with precancerous lesions or cancers associated with infection by HPV, as metabolic reprogramming is an essential hallmark in carcinogenesis. Likewise, the enzymes or proteins involved in energy metabolism affected by these variants could be studied as possible therapeutic targets or diagnostic and prognostic markers in cancers associated with HPV infection.

## Figures and Tables

**Table 1:** Effect of HPV 16 E6 and E7 variants on metabolic reprogramming in cancer.

Oncoprotein	Sublineage	Nucleotide or Protein Variant	Effect on Protein or Signaling Pathway	Effect on Metabolism	Reference
E6	Europeo	E-prototype	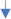 p53	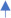 glycolysis	[108,109,110,111,112]
		R17I	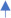 c-Myc expression and activation	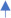 glycolytic metabolism 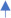 nucleotide synthesis 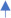 glutaminolysis	[107,132,133,134,135,136]
		Q21D			
		E-A126 (R8Q)	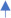 c-Myc expression and activation 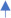 Wnt/β-catenin signaling pathway activation	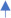 glycolytic metabolism 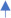 nucleotide synthesis 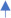 glutaminolysis 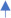 Warburg effect 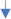 mitochondrial respiration	[107,132,133,134,135,136,154]
		E-G131 (R10G)	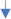 p53 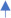 Wnt/β-catenin signaling pathway activation	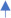 glycolysis 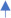 Warburg effect 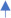 mitochondrial respiration	[108,109,110,111,112,135,136,154]
		E-T245 (R48W)	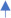 Wnt/β-catenin signaling pathway activation	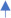 glycolytic metabolism 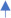 Warburg effect 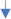 mitochondrial respiration	[135,136,154]
		E-G350 (L83V)	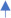 MAPK signaling pathway activation 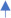 Wnt/β-catenin signaling pathway activation	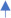 autophagy 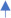 glycolytic metabolism 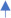 Warburg effect 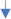 mitochondrial respiration	[135,136,144,150,151,154]
	Asian	As (D25E)	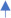 HIF-1α stability 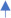 STAT3 expression 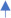 miR-21 expression 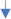 AIFM2 expression 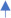 ENO1 expression cytochrome liberation 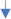 glutathione S transferase Pi 1 (GSTP1) levels	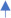 Warburg effect 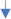 mitochondrial respiration 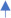 fatty acid synthesis 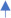 lipid metabolism 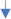 oxidative phosphorylation 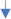 phase II detox metabolism	[13,16,17,137,139,140,141,142,158,159,160,168]
	Asian-American	AA (Q14H/H78Y/L83V)	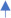 HIF-1α stability 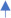 STAT3 levels 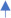 MAP2K1 expression 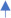 MAPK-extracellular signal-regulated kinase (ERK), MAPK-p38 y PI3K/AKT activation 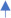 mTOR activation 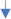 IDH1 IDH2 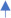 GAPDH and PKM2 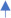 phosphoglucomutase and aldoketoreductase 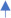 CAIX expression PRKAG2 interaction 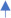 SLC26A2 expression 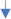 p53	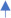 autophagy 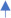 glucose uptake 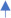 glycolytic metabolism and Warburg effect 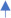 internalization of glucose into the cell 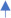 oxalate levels, mitochondrial damage	[6,13,16,17,108,109,110,111,112,137,139,140,141,142,145,149,150,151,163,171]
	African	Afr2a(R10I/Q14D/H78Y)	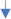 p53	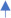 glycolysis	[108,109,110,111,112]
E7		A4 (N29S)	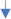 pRb	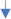 oxidative metabolism 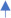 glutaminolysis 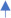 nucleotide synthesis	[88,128,129,130]
		A5 (L28F)			

Up or down arrows indicate the increase or decrease, respectively.
